# Association between Polyunsaturated Fatty Acid Profile and Bronchial Inflammation in Bronchiolitis Obliterans

**DOI:** 10.1155/2023/3406399

**Published:** 2023-07-05

**Authors:** Silvija P. Jerkic, Laura Bächle, Ruth Pia Duecker, Lucia Gronau, Andreas G. Chiocchetti, Stefan Zielen, Ralf Schubert

**Affiliations:** ^1^Division of Allergy, Pulmonology and Cystic Fibrosis, Department for Children and Adolescents, Goethe University, Frankfurt, Germany; ^2^Department of Food Technology, University of Applied Science, Fulda, Germany; ^3^Department of Child and Adolescent Psychiatry, Psychosomatics and Psychotherapy, University Hospital Frankfurt, Goethe-University, Frankfurt am Main 60590, Germany

## Abstract

**Introduction:**

Bronchiolitis obliterans (BO) is a chronic lung disease, which occurs after an insult to the lower airways, in particular after airway infections or after stem cell transplantation, and which results in persistent inflammation. *N*–3 and *n*–6 polyunsaturated fatty acids (PUFA) have been shown to influence the inflammatory processes in chronic inflammatory conditions. Since BO is maintained by persistent pulmonary inflammation, a disbalanced *n*–6/*n*–3 fatty acid profile could support the inflammatory process in patients with BO and therefore, could become an approach to new therapeutic options.

**Methods:**

Twenty-five patients with BO (age: 13; 7–39) and 26 healthy controls (age: 19; 7–31) participated in the study. Lung function (forced viral capacity (FVC), forced expiratory volume 1 (FEV1), residual volume (RV)), and lung clearance index (LCI) were measured. Induced sputum was analyzed for cytology and cytokine levels (IL-1*ß*, IL-6, IL-8, TNF-*α*) using cytometric bead array (CBA). The PUFA profile was determined in the serum and induced sputum by gas chromatography.

**Results:**

Patients presented with significantly lower FVC and FEV1 as well as higher RV and LCI measurements compared to the control group. Apart from a massive airway inflammation indicated by elevated numbers of total cells and neutrophils, the CBA analysis showed significantly increased levels of IL-1*β*, IL-6, and IL-8. The analysis of PUFA in sputum and serum revealed a significant difference in the ratio between the *n*–6 PUFA arachidonic acid (AA) and the *n*–3 PUFA docosahexaenoic acid (DHA) (AA : DHA). Furthermore, the AA : DHA ratio significantly correlated with the inflammatory cytokines in induced sputum.

**Conclusion:**

Lung function in BO is significantly impaired and associated with uncontrolled neutrophil-dominated airway inflammation. Furthermore, the imbalance in the AA/DHA ratio in favor of *n*–6 PUFA demonstrates a pro-inflammatory microenvironment in the cell membrane, which correlates with the inflammatory cytokines in induced sputum and might be an option for an anti-inflammatory therapy in BO.

## 1. Introduction

Bronchiolitis obliterans (BO) is a rare, chronic disease that originated from an initial insult to the small airways [1]. Such insult to the lower airways can be caused by pathogens such as adenovirus, influenza, respiratory syncytial virus, or mycoplasma pneumonia. [2, 3]. Previous studies reported patients with BO mainly among certain populations in South America, Argentina [4], North America, and Canada [5]. However, the interest in BO is growing and there are various studies from other countries [6–8] reporting cohorts of patients with BO. In view of this variable prevalence, there may be a genetic predisposition [9]. In addition, local nutrition and differences in serotypes of the causative pathogens are likely to influence the development of BO. [10].

As outlined by Jerkic et al. [10], BO is considered to be a rare disease that is characterized by nonreversible airway obstruction with functional and radiological evidence of small airway disease. Since the nomenclature is very different, the incidence of BO is likely more frequent than expected. After the causative insult, patients with BO present clinically with persistent tachypnea, crackles, wheezing, and hypoxemia [11]. Teper et al. [12] described that there is a fixed airway obstruction with decreased forced expiratory volume 1 (FEV1), reduced FEV1/forced viral capacity (FVC) ratio, and reduced end-expiratory flow (MEF25) in pulmonary function tests (PFTs) [10, 12, 13]. Hyperinflation is indicated by an increased residual volume (RV) and an increased ratio of RV to total lung capacity (TLC) in PFTs (RV/TLC). In addition, patients with BO respond little or not at all to bronchodilation [10]. Eber et al. [14] described that the pathognomonic finding in chest high-resolution computed tomography (HRCT) in BO is a mosaic attenuation, which is a variation in the density of alveolar lung tissue that occurs from alveolar hyperinflation and hypoxic vasoconstriction of the effected lungs [10].

Previous studies showed that after an initial insult, there is a severely altered repair process to the lower airways [10], indicating that persistent inflammation seems to play an important role in the disease process. In sputum samples of patients with BO, Eckrich et al. [8] revealed an increased number of neutrophils persisting over a period of 4 weeks. Furthermore, studies from different centers demonstrated that persistent neutrophilic inflammation is present even several years after disease onset [13, 15]. It seems that the inflammatory process caused by the initial insult persists, and the natural mechanism of the following resolution remains hindered.

Epidemiological studies suggested a correlation between inflammatory diseases and nutrition, showing that diseases such as coronary artery disease or bronchial asthma occur less frequently in populations with an omega-3 FA (fatty acid) rich diet intake [16, 17]. As part of our food, fatty acids not only contribute to energy production, but they also play an important role in cell structure and as messenger substances in physiological processes. As energy source, they are stored in the form of triacylglycerols while they are built into cell membranes as components of phospholipids or glycolipids. Polyunsaturated fatty acids (PUFAs) have more than one double bond in their chain of carbon atoms and have the potential to modify inflammatory processes [18]. While pro-inflammatory mediators mainly derive from *n*–6 PUFAs such as arachidonic acid (AA), the *n*–3 PUFAs, especially eicosapentaenoic acid (EPA) and docosahexaenoic acid (DHA) evolve into “specialized pro-resolving mediators” (SPM), such as resolvines, protectines, and maresines, which have potent anti-inflammatory and inflammation capacity [19–21].

Studies in patients with chronic lung diseases, such as cystic fibrosis (CF) and asthma, have shown a shift in the fatty acid profile in favor of *n*–6 PUFA [22, 23], which suggests that changes in concentration and ratio of *n*–3 PUFA to *n*–6 PUFA contribute to persistent pulmonary inflammation and hinder the resolution of the inflammatory process [24–27].

In view of BO being maintained by persistent inflammation in the small airways, the aim of this study was to investigate the fatty acid profile in serum and induced sputum in a cohort of patients with BO and to analyze the role of PUFA in the pulmonary inflammatory process of BO.

## 2. Methods

### 2.1. Patients and Controls

In this prospective cohort study, 25 patients, who have been diagnosed with BO at the Division of Allergy, Pulmonology and Cystic Fibrosis at the Children's University Hospital Frankfurt/Main, Germany, were included. The Children's University Hospital is a tertiary center associated with the Goethe University Frankfurt and takes referrals from all over the country. All 25 patients were identified by the register of our specialized clinic for rare lung diseases and were known to the authors. In addition, 26 healthy controls were recruited randomly. Blood samples were drawn in nonfasting condition by venopuncture into ethylenediaminetetraacetic acid tubes the same day as the induced sputum was obtained.

As described in previous studies, BO was defined as follows: (1) history of severe respiratory infection, (2) persistent symptoms such as tachypnea, cough, wheezing, exercise intolerance, and hypoxemia, (3) impaired lung function with evidence of airway obstruction with FEV1 <75% without reversibility, and (4) mosaic attenuation, air trapping, and bronchial wall thickening on chest HRCT [15].

The diagnostic criteria of BO were defined clinically as follows: history of severe chest infection, persistent airway obstruction, unresponsiveness to bronchodilator treatment, and radiological evidence of mosaic attenuation, hyperinflation, and bronchial wall thickening on chest HRCT [10].

### 2.2. Pulmonary Function Test

As described in previous studies, PFTs (spirometry and body-plethysmography) were performed by body plethysmograph (Vyntus™, Vyaire, Höchberg, Germany) according to the recommendations of the European Respiratory Society [15, 28] and the following measurements were obtained: FVC, FEV1, FEV1/FVC, RV, and RV/TLC.

The lung clearance index (LCI) was analyzed by EasyOne Pro Lab (ndd Medical Technologies, Andover, Massachusetts, USA) as described recently [15, 29].

### 2.3. Airway Reversibility

Bronchodilator reversibility was determined after inhalation of 400 *µ*g of salbutamol via metered dose inhaler with a spacer. Reversibility was defined as yes/no using *a* ≥ 12% and 200 mL post-bronchodilator change as described recently [15, 29].

### 2.4. Sputum Collection and Cell Analysis

Sputum collection and cell analysis were performed as previously described [29–31]. Sputum was obtained by stepwise inhalation of 3%, 4%, and 5% saline solution every 7 min. To overcome the possible risk of severe bronchoconstriction, 200 *µ*g (two puffs) of salbutamol were given before sputum induction. In addition, we performed spirometry after every inhalation step with concentrations of 3%, 4%, and 5%. If there was a drop of FEV1 > 12%, the sputum induction was discontinued, and salbutamol was inhaled immediately.

After quantification, sputum plugs were selected from the obtained samples, 4× (weight/volume) dithiothreitol (DTT, 0.1%) was added, and samples were incubated on ice for 15 min. Subsequently, 2× weight/volume of phosphate-buffered saline was added, and samples were centrifuged for 10 min at 790 × *g*. Supernatants were collected and stored at –80°C until further protein analyses. The pellets were resuspended, and the slides were prepared by cytospin centrifugation (Shandon Cytospin 3 Centrifuge). Four hundred cells per slide were counted and quantified for neutrophils, lymphocytes, eosinophils, and macrophages using the Leucodiff 800plus instrument (Instrumentation Laboratory, Bedford, MA, USA).

### 2.5. Cytometric Bead Array (CBA)

The concentration of the cytokines, IL-1*β*, IL-6, and IL-8, was analyzed in sputum supernatants using BD™ CBA Flex Set System (BD Bioscience-PharMingen, San Diego, CA, USA) according to the manufacturer's instructions [29, 31]. DTT (0.025%) was added to the standard curve and enzyme immunoassay buffer [32]. The lower detection limits of the cytokines were 2.3, 1.2, and 1.6 pg/mL for IL-1*β*, IL-8, and IL-6, respectively.

### 2.6. Measurement of Fatty Acids from Serum and Sputum

The measurement was carried out at the University of Applied Sciences in Fulda. The PUFA levels were determined by fatty acid methyl ester (FAME) from the cell pellet of the sputum and from erythrocyte membranes by capillary gas chromatography according to the method of Beermann et al. [33]. FAMEs were extracted from the total lipids of membranes, and an aliquot of 3 mg of total lipid extract was resolved in 2 mL of methanol/hexane (v/v) 4 : 1 plus pyrogallol and was methylated with 200 *µ*L of acetyl chloride at 100°C for 1 hr, for derivatization. Afterward, 5 mL of 6% K_2_CO_3_ were added, and samples were centrifuged for 10 min at 2,200 × *g*. The upper hexane phase containing the FAME was dried with Na_2_SO_4_. Finally, the FAMEs were analyzed by capillary gas–liquid chromatography on a LS 32 Varian Chrompack system (Varian Chrompack, Middelburg, The Netherlands) [34].

A table with all fatty acids analyzed in sputum and blood, including saturated and monounsaturated fatty acids, is included within the supplement (Tables S2 and S3).

In addition, to provide an estimation for *n*–6 and *n*–3 PUFA distribution, the ratio between AA and DHA (AA : DHA) was calculated.

### 2.7. Statistical Analysis

The data analysis was performed using GraphPad Prism 9.3 (Graph Pad Software Inc., La Jolla, CA, USA) and Microsoft Excel. The Kruskal–Wallis test or the Mann–Whitney test were used to analyze group differences between BO patients and control subjects depending on the normality and homogeneity of variance assumptions, respectively. For correlation analysis, the Spearman or the nonparametric Pearson test was used. Statistical significance was assumed at *p* < 0.05.

## 3. Results

As shown in Table 1, we enrolled 25 patients (median age 13, range 7–39, male/female ratio 19/6). We compared the patient's data with those of 26 healthy control subjects (median age 19, range 7–31; male/female ratio 14/12) (Table 1).

### 3.1. Pulmonary Function

As shown before, measurement of lung function parameters showed a significant reduction of FVC (patients 71.7%, 31.2–90.4; controls 95.1%, 73.9–121.1; *p* < 0.001), of FEV1 (patients 48.0%, 26.3–82.9; controls 95.5%, 76.7–124.2; *p* < 0.001) and FEV1/FVC (patients 68.3; 42.1–95.1; controls 89.7, 52.6–115.4; *p* < 0.001) as well as a significant increase of RV (patients 236.1%, 107.0–313.2; controls 171.0%, 92.9–274.5; *p* < 0.01) among patients with Postinfectious bronchiolitis obliterans (PiBO) compared to controls.

**Figure 1:**
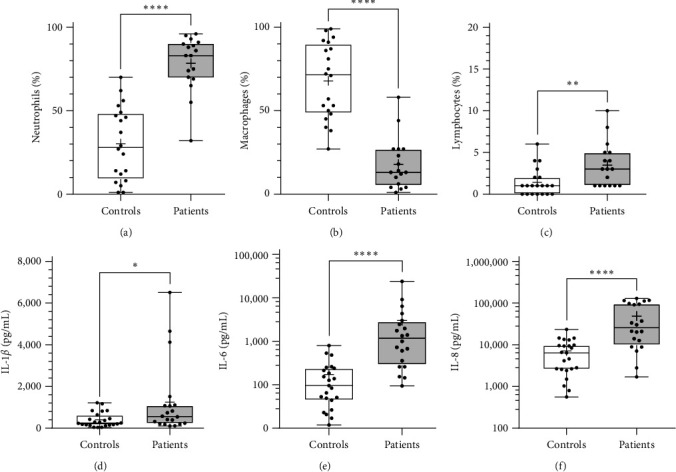
Bronchial inflammation. Inflammation in the induced sputum was determined by the percentage of (a) neutrophils, (b) macrophages, and (c) lymphocytes by microscopic differentiation of the cells after Giemsa staining in samples from BO patients (*n* = 20) and healthy controls (*n* = 23). In addition, flow cytometric analysis of the cytokines (d) IL-1b, (e) IL-6, and (f) IL-8 using a cytometric bead array was performed.  ^*∗*^*p* < 0.05,  ^*∗∗*^*p* < 0.01,  ^*∗∗∗∗*^*p* < 0.0001.

There was a significant difference in the LCI measurements between the patient and control group (*p* < 0.001). The patient group presented with an increased LCI of 13.33 (range 7.84–20.21), while the control group presented with a regular LCI of 7.94 (range 6.1–11.9) (Figure 1, Table S1).

### 3.2. Bronchial Inflammation

The sputum samples of the BO group revealed a significantly higher percentage of neutrophils compared to the control group (patients 83%, 32–96; controls 28%, 1–70; *p* < 0.0001). The median proportion of alveolar macrophages was 13% (1–58) in the patient group and thus, significantly lower than the proportion of the alveolar macrophages in the control group (72%, 27–99; *p* < 0.0001). Interestingly, percentages of lymphocytes were significantly higher in the BO group (patients 3%, 1–10; controls 1%, 0–6; *p* < 0.01). (Figure 1(a)–1(c)). No differences in the percentage of eosinophils and basophils could be detected between the groups.

The inflammatory cytokines such as IL-1*β* (patients 556 pg/mL, 106–6,511; controls 251 pg/mL, 36.5–1,218; *p* < 0.05), IL-6 (patients 1,166 pg/mL, 94.0–23,939; controls 95.2 pg/mL, 11.8–797; *p* < 0.0001) and IL-8 (patients 25,579 pg/mL, 1,698–129,814; controls 6,387 pg/mL, 560–23,302; *p* < 0.0001) were significantly increased in the patient group, compared to controls (Figures 1(d) and 1(e)).

### 3.3. PUFA Levels in Sputum and Blood Cells

**Figure 2:**
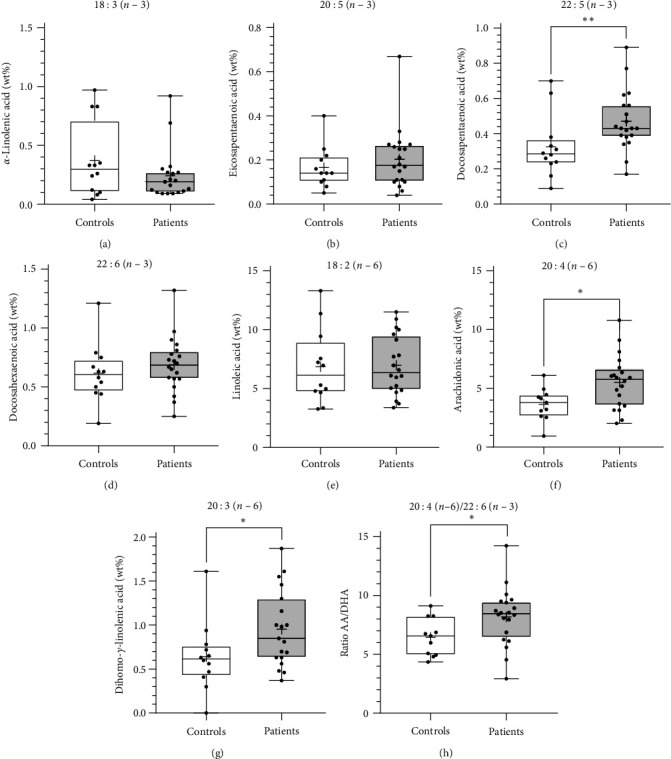
Cellular LCPUFA concentrations in sputum cells. LCPUFA analysis of (a) alpha-linolenic acid (ALA), (b) eicosapentaenoic acid (EPA), (c) docosapentaenoic acid (*n*–3 DPA), (d) docosahexaenoic acid (DHA), (e) linoleic acid (LA), (f) arachidonic acid (AA), and (g), dihomogammalinolenic acid (DGLA) was determined from the cell pellet of the sputum derived from BO patients (*n* = 19) and healthy controls (*n* = 12) by capillary gas chromatography. In addition, (h) the ratio between AA and DHA (AA : DHA), which provides an estimation for omega-6 and omega-3 fatty acid distribution, was calculated.  ^*∗*^*p* < 0.05,  ^*∗∗*^*p* < 0.01.

In sputum, levels of DPA (patients 0.43 wt%, 0.17–0.89; controls 0.30 wt%, 0.09–0.70; *p* < 0.01), AA (patients 5.89 wt%, 2.02–10.8; controls 3.97 wt%, 2.61–6.11; *p* < 0.05) and dihomogammalinolenic acid (patients 0.85 wt%, 0.37–1.87; controls 0.62 wt%, 0.39–1.61; *p* < 0.05) was significantly increased in BO patients (Figure 2). Accordingly, the AA : DHA ratio was significantly higher in the BO group (patients 8.39, 2.94–14.23; controls 6.28, 4.36–9.12; *p* < 0.05) (Figure 2, Table S2).

**Figure 3:**
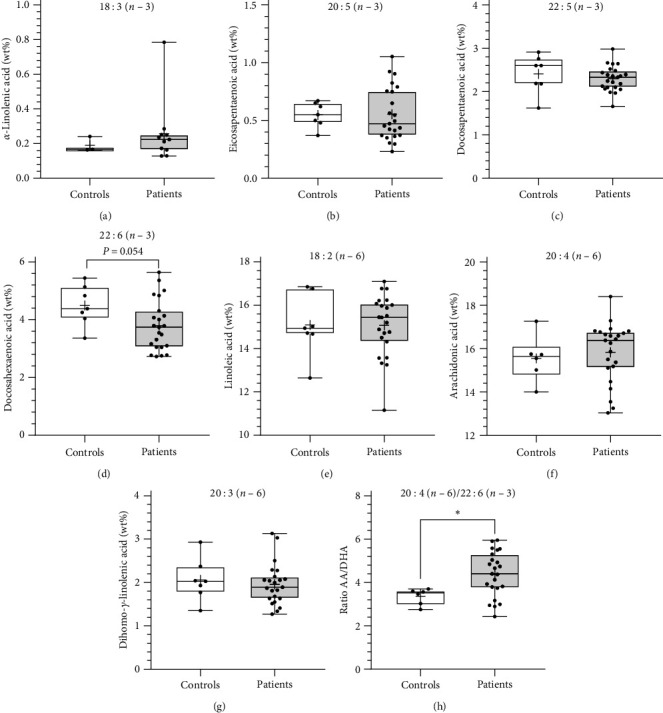
Cellular LCPUFA concentrations in blood cells. LCPUFA analysis of (a) alpha-linolenic acid (ALA), (b) eicosapentaenoic acid (EPA), (c) docosapentaenoic acid (*n*–3 DPA), (d) docosahexaenoic acid (DHA), (e) linoleic acid (LA), (f) arachidonic acid (AA), and (g) dihomogammalinolenic acid (DGLA) was determined from the cell pellet of the sputum derived from BO patients (*n* = 19) and healthy controls (*n* = 12) by capillary gas chromatography. In addition, (h) the ratio between AA and DHA (AA : DHA), which provides an estimation for omega-6 and omega-3 fatty acid distribution, was calculated.  ^*∗*^*p* < 0.05,  ^*∗∗*^*p* < 0.01.

**Figure 4:**
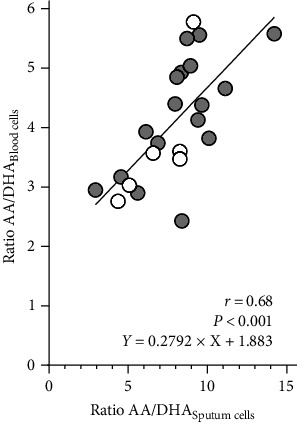
Comparison of AA : DHA ratios between sputum and blood. Correlation is shown between the ratio AA and DHA (AA : DHA) calculated for sputum and blood. Correlation coefficient and *p*-value are given in the figure.

In serum, levels of EPA (patients 0.47 wt%, 0.23–1.05; controls 0.55 wt%, 0.37–0.67; *p* = 0.0522) showed a trend to be lower in BO patients and, as shown in the sputum the AA : DHA ratio was significantly higher in the BO group compared to the control group (patients 4.40, 2.43–5.96; controls 3.52, 2.76–3.70; *p* < 0.05). The other fatty acids did not show any difference between patients and controls, neither in sputum nor in blood (Figure 3, Table S3). There was a significant correlation between the AA : DHA ratio from the sputum and the blood cells (*r* = 0.68; *p* < 0.001; Figure 4). Our analyses also showed that gender had no significant effect on the AA/DHA ratio.

### 3.4. Correlations between the AA : DHA Ratio, Lung Function Parameters, and Inflammation

**Figure 5:**
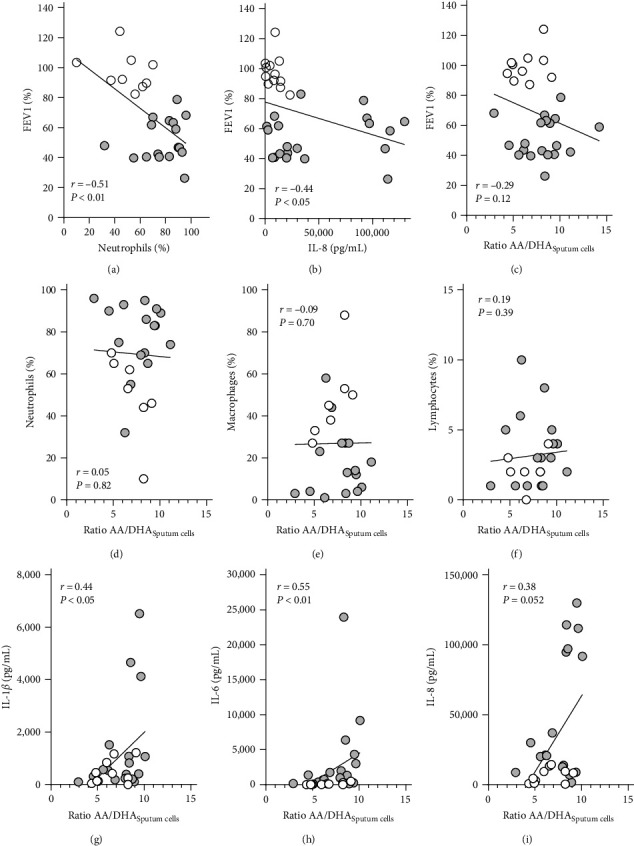
Correlations between the AA : DHA ratio, lung function parameters, and inflammation. Correlations were calculated between FEV1 and (a) neutrophils, (b) IL-8, and (c) the ratio between AA and DHA (AA : DHA), between the AA : DHA ratio and the percentage of (d) neutrophils, (e) macrophages, and (f) lymphocytes as well as between the AA : DHA ratio and (g) Il-1b, (h) IL-6, and (i) IL-8 in induced sputum. Correlation coefficient and *p*-value are given in the figure.

Correlation analyses were carried out to examine the extent to which the AA : DHA ratio is related to lung function and inflammation. FEV1 significantly correlated with the percentage of neutrophils (*r* = –0.51; *p* < 0.01) and with the IL-8 concentration (*r* = –0.44; *p* < 0.05) concentration, but not with the AA : DHA ratio (Figure 5(a)–5(c)). This was also the case for the other lung function parameters, with the exception of the RV, which showed no correlation with the cytokines (Table S1). Interestingly, the LCI showed a strong, though not significant, trend toward correlation with the fatty acid ratio.

While the sputum neutrophils, macrophages, and lymphocytes showed no correlation with the AA : DHA ratio, IL-1*β* (*r* = 0.44; *p* < 0.05) and IL-6 (*r* = 0.55; *p* < 0.01), correlated significantly and IL-8 (*r* = 0.38; *p* = 0.0516) showed a strong tendency with the fatty acid ratio.

## 4. Discussion

The pathogenesis of BO is based on persistent inflammation in the small airways following a variety of inciting diseases and proceeds into an inadequate resolution of the inflammatory process [10, 35]. The persistent influx of neutrophil granulocytes and the release of inflammatory mediators leads to fibrotic tissue remodeling and obstruction of the bronchioles resulting into small airway disease [8, 29, 36–38]. Clinically, patients with BO present with significant flow limitations, particularly affecting the small airways and typical signs of air trapping in distal areas of the lung on HRCT chest. According to previous studies, there was a correlation between the neutrophil count in sputum and bronchoalveolar lavage (BAL) fluid and obstruction of the small airways [8, 29, 38]. This indicates that there is a clear relationship between persistent neutrophilic inflammation and small airway disease [8, 26, 39]. These findings were replicated by the analysis of the sputum samples in our BO cohort, which confirmed that the number of neutrophils remains significantly higher in the affected patients compared to healthy controls.

Additionally, further analysis of the sputum samples confirmed significantly increased levels of the pro-inflammatory markers IL-1*β*, IL-6, as well as IL-8, which clearly points out a chronic inflammatory process in the lung. These findings are in line with a previous study of our group, revealing that the levels of IL-1*β*, IL-6, and IL-8 were higher in BO patients compared to controls [8, 29, 38].

The chronic inflammatory process of the small airways in BO has been described previously. However, to date, it remains unclear what maintains the persistent inflammatory process and what may hinder a well-ordered resolution. Unsaturated fatty acids and their derivates play a significant role in the regulation of various chronic inflammatory processes [21, 24–26].

While *n*–6 PUFA and their derivates have been shown to maintain the inflammatory process, *n*–3 PUFA and their DHA derivates D-resolvins, protectins, and maresins have been shown to promote anti-inflammatory effects and to initiate the process of resolution in the inflammatory event [18–21]. Duvall and Levy [23] have shown that the DHA derived SPMs influence pro-resolving pathways by disrupting the migration of neutrophil granulocytes into the tissue, initiating the apoptosis and phagocytosis of involved neutrophils and by suppressing proinflammatory mediators.

For the first time, fatty acid profiles in BO were determined and compared with fatty acid profiles of healthy controls. There was a significant increase of AA and a decrease of DPA in the sputum of the BO group. Furthermore, a shifted ratio of AA : DHA in favor of *n*–6 PUFA was observed. These findings indicate that the increased amount of *n*–6 PUFA creates a pro-inflammatory environment, which consequently sustains the persistent inflammation in BO. Similar findings have been shown in patients with other chronic inflammatory diseases such as CF [23, 36, 39]. As in BO, the role of chronic neutrophilic inflammation in the small airways of patients with CF has been described previously [32, 39, 40]. Fatty acid analysis in patients with CF revealed a significantly increased ration of AA : DHA in nasal and rectal biopsies, which suggests a correlation between fatty acid profile and chronic inflammatory process [26, 40].

In addition to the PUFA analysis in sputum, the determination of PUFA in serum was performed. As in sputum analysis, there was a significant difference in the AA : DHA ratio between the patients and the healthy controls. Analogous to the sputum analysis, a ratio shift in favor of *n*–6 PUFA was found in the serum of the patients with BO. Comparable results were found in the serum of patients with CF and asthma. Children with asthma as well as CF showed an increased ratio shifted in favor of *n*–6 PUFA in serum samples [26, 38, 40]. The findings in our study suggest a correlation between the changes in the fatty acid profile in sputum and serum samples.

A possible reason for the reduced PUFA *n*–3 levels could be explained by the persistently severe inflammation and the associated release of EPA, DPA *n*–3, and DHA. The inflammation is too high to be controlled, and the body has decreased its level of *n*–3 LCPUFA. Another reason could be related to a different dietary intake of PUFA in patients with BO versus healthy subjects or to a deficiency of replenishing the *n*–3 PUFA reservoir. There is evidence that reduced plasma DHA levels in patients with CF may be due to suboptimal intestinal absorption of fatty acids [40].

Next, we examined the relationship between the AA : DHA ratio and inflammation. Although we did not find a correlation between neutrophils in the sputum and the fatty acid ratio, our analysis showed a significant correlation or at least a strong tendency to the concentrations of the inflammatory cytokines IL-1*β*, IL-6, and IL-8, respectively. Thus, our data revealed that the cellular PUFA composition has an influence on the release of soluble inflammatory mediators rather than on the diapedesis of neutrophils. Fussbroich et al. [41] also demonstrated that a combination of PUFA reduces the expression of inflammatory mediators in the airways of asthmatic mice, including IL-6. However, the number of neutrophils in the BAL was not affected. As precursors of highly potent lipid mediators, PUFA play an important role in inflammatory processes and in controlling the inflammatory resolution on cellular level in the broncho-alveolar system [42].

Interestingly, sputum samples of patients with CF revealed a significantly decreased concentration of the pro-resolving lipid mediator Resolvin D1 indicating a pro-inflammatory environment [40]. However, mediators deriving from PUFA were not measured in this study. However, in view of the relative deficiency of DHA, we presume a decreased concentration of pro-resolving lipid mediators, SPMs, in patients with BO. Further analysis of resolvins, maresins, and protectins in sputum samples of patients with BO are necessary to confirm this hypothesis.

Treatment in BO is empirical and scarce, and there is no accepted treatment protocol. Jerkic et al. [10] described that the fluticason, azithromycin, and montelukast regime in combination with steroid pulses at the start of the therapy slows down the inflammatory process and prevents from further decline in lung function. To date, there is no satisfactory therapeutic option known which would disrupt the neutrophilic inflammation and hinder the progression into irreversible, fibrotic changes of the airway epithelium, which subsequently ends in a fixed, mixed pattern expressed on PFTs.

A balanced ratio of *n*–6 : *n*–3 PUFA has been described to have beneficial effects on disease progression in chronic inflammatory conditions [22]. In chronic inflammatory conditions such as asthma and CF, some therapeutic benefit of *n*–3 PUFA substitution has been demonstrated [18, 27]. In patients with CF, an 8-monthly oral intake of *n*–3 PUFA led to decreased inflammatory parameters and an improvement in FEV1 [43]. In addition, a reduction in pulmonary exacerbations and fewer antibiotic treatments were observed [43, 44]. These findings suggest that patients with BO are likely to benefit similarly from a substitution of *n*–3 PUFA.

In conclusion, the analysis of PUFA in sputum and serum of patients with BO revealed a significant difference in the AA :DHA ratio in patients with BO leading to a shift in favor of *n*–6 PUFA, which promotes a pro-inflammatory environment. A therapeutic shift in favor of the anti-inflammatory *n*–3 PUFA and pro-resolving lipid mediators to dampen pathological inflammation are likely to have a beneficial health impact.

## Figures and Tables

**Table 1:** Patient characteristics.

	Controls	Patients	*p*-Value
Number	26	25	
Sex m : f	14 : 12	19 : 6	
Age	19	13	
	(7–31)	(7–39)	
FVC (%)	95.1	71.7	*p* < 0.001
	(73.9–121.1)	(31.2–90.4)	
FEV1 (%)	95.5	48.0	*p* < 0.001
	(76.7–124.2)	(26.3–82.9)	
FEV1/FVC (%)	89.7	68.3	*p* < 0.001
	(52.6–115.4)	(42.1–95.1)	
RV (%)	171.0	236.1	*p* < 0.01
	(92.9–274.5)	(107.0–313.2)	
LCI	7.94	13.33	*p* < 0.001
	(6.1–11.9)	(7.4–20.2)	

Data are shown as median and range. Group differences between BO patients and control subjects were analysed using the Kruskal–Wallis test or Mann–Whitney test depending on the normality and homogeneity of variance assumptions. Abbreviations: FVC, forced viral capacity; FEV1, forced expiratory volume; RV, residual volume; LCI, lung clearance index.

## Data Availability

The data used to support the findings of this study are available from the corresponding author upon request.

## Supplementary Materials

Supplementary Materials Table S1: correlations between the AA : DHA ratio, lung function parameters and inflammation. Table S2: analysed fatty acids in sputum cells. Table S3: analysed fatty acids in blood cells.
