# Supplementary material for: Association between Polyunsaturated Fatty Acid Profile and Bronchial Inflammation in Bronchiolitis Obliterans
Source: Mediators Inflamm. 2023 Jul 5;2023:3406399. doi: 10.1155/2023/3406399 (PMC10338129; doi:10.1155/2023/3406399)
Supplement: Supplementary Materials — Table S1: correlations between the AA : DHA ratio, lung function parameters and inflammation. Table S2: analysed fatty acids in sputum cells. Table S3: analysed fatty acids in blood cells. [file 3406399.f1.pdf]

Suppl. Table 1. Correlations between the AA:DHA ratio, lung function parameters and inflammation

1. Parameter	2. Parameter	*r*	*p*-Value
FVC	Neutrophils	-0.48	<0.05
FVC	Macrophages	0.49	<0.05
FVC	Lymphocytes	0.05	0.82
FVC	IL-1β	-0.32	0.08
FVC	IL-6	-0.59	<0.01
FVC	IL-8	-0.57	<0.001
FVC	AA:DHA	-0.25	0.19
FEV1	Neutrophils	-0.51	<0.01
FEV1	Macrophages	0.53	<0.01
FEV1	Lymphocytes	0.01	0.97
FEV1	IL-1β	-0.20	0.28
FEV1	IL-6	-0.55	<0.01
FEV1	IL-8	-0.44	<0.05
FEV1	AA:DHA	-0.29	0.12
RV	Neutrophils	0.45	<0.05
RV	Macrophages	-0.46	<0.05
RV	Lymphocytes	0.04	0.86
RV	IL-1β	-0.13	0.50
RV	IL-6	0.30	0.11
RV	IL-8	0.13	0.47
RV	AA:DHA	0.13	0.52
LCI	Neutrophils	0.67	<0.01
LCI	Macrophages	-0.99	<0.0001
LCI	Lymphocytes	0.20	0.32
LCI	IL-1β	0.33	0.11
LCI	IL-6	0.20	0.34
LCI	IL-8	0.52	<0.01
LCI	AA:DHA	0.37	0.08
Neutrophils	AA:DHA	0.05	0.82
Macrophages	AA:DHA	-0.09	0.70
Lymphocytes	AA:DHA	0.19	0.39
IL-1β	AA:DHA	0.44	<0.05
IL-6	AA:DHA	0.54	<0.01
IL-8	AA:DHA	0.38	<0.05

Suppl. Table 2. analysed fatty acids in sputum cells

Fatty acids %wt	Patients (Mean)	Patients (SD)	Controls (Mean)	Controls (SD)
C8:0	0.26	0.48	0.08	0.08
C10:0	0.17	0.35	0.06	0.07
C11:0	0.48	0.72	0.16	0.15
C12:0	0.17	0.29	0.05	0.04
C13:0	0.15	0.24	0.04	0.05
C14:0	0.24	0.46	0.04	0.02
C14:1	0.24	0.19	0.28	0.14
C15:0	0.67	0.19	0.86	0.78
C15:1	0.23	0.38	0.03	0.02
C16:0	44.56	9.43	42.64	11.89
C16:1n7t	0.20	0.28	0.19	0.18
C16:1n7c	2.04	0.73	2.23	1.30
C17:1	0.03	0.01	0.14	0.11
C18:0	7.05	5.83	11.24	6.12
C18:1n9t	0.32	0.19	0.53	0.34
C18:1n9c	22.74	3.13	23.40	8.64
C18:1n7 (VAC)	1.93	0.59	6.40	15.02
C18:2n6t (LEA)	0.08	0.03	0.17	0.13
C18:2n6c (LA)	6.97	2.52	6.85	3.15
C18:3n6 (GLA)	1.26	1.51	1.73	3.06
C18:3n3 (ALA)	0.25	0.22	0.37	0.32
C20:0	0.20	0.14	0.31	0.20
C18:4n3 (STA)	0.09	0.15	0.31	0.54
C20:1n9c	0.44	0.28	0.30	0.15
C21:0	0.05	0.07	0.13	0.35
C20:2	0.26	0.17	0.20	0.11
C20:3n9 (MA)	0.15	0.05	1.55	4.21
C20:3n6	0.92	0.44	0.69	0.39
C20:4n6 (AA)	5.51	2.26	3.64	1.38
C20:3n3	0.06	0.06	0.06	0.04
C22:0	0.08	0.04	0.17	0.10
C22:1n9	0.13	0.12	0.09	0.04
C20:5n3 (EPA)	0.20	0.14	0.17	0.09
C23:0	0.06	0.11	0.02	0.02
C22:2	0.03	0.02	0.03	0.03
C22:4n6 (ADA)	0.80	0.43	0.43	0.20
C24:0	0.10	0.07	0.17	0.12
C24:1n9	0.09	0.04	0.06	0.02
C22:5n3 (DPA)	0.47	0.17	0.32	0.18
C22:6n3 (DHA)	0.69	0.23	0.62	0.25

Data is shown as Mean ±SD.

Suppl. Table 3. analysed fatty acids in blood cells

Fatty acid %wt	Patients (Mean)	Patients (SD)	Controls (Mean)	Controls (SD)
C14:0	0.37	0.29	0.34	0.09
C15:0	0.14	0.02	0.18	0.04
C16:1n7t	21.14	3.22	22.57	1.77
C16:1n7c	0.48	0.20	0.58	0.40
C17:0	0.30	0.15	0.28	0.04
C18:0	16.22	1.13	15.20	1.74
C18:1n9c	16.22	1.39	15.28	1.30
C18:1n7	1.33	0.18	1.43	0.20
C18:2n6c	15.10	1.46	15.09	1.43
C18:3n6	0.17	0.10	n.d.	n.d.
C18:3n3	0.26	0.18	0.19	0.04
C20:1n9c	0.31	0.12	0.27	0.05
C20:2	0.29	0.19	0.24	0.03
C20:3n9	0.12	0.02	n.d.	n.d.
C20:3n6	1.96	0.48	2.06	0.49
C20:4n6	15.83	1.38	15.55	1.06
C22:0	0.12	0.03	n.d.	n.d.
C22:1n9	0.24	0.17	0.22	0.05
C20:5n3	055	0.23	0.55	0.10
C22:4n6 (ADA)	3.99	3.13	3.09	0.44
C22:5n3	2.30	0.29	2.41	0.44
C22:6n3	3.80	0,87	4.49	0.70

Data is shown as Mean ±SD. n.d., not defined
